# Diagnostic prediction of urinary [TIMP-2] x [IGFBP7] for acute kidney injury: A meta-analysis exploring detection time and cutoff levels

**DOI:** 10.18632/oncotarget.21903

**Published:** 2017-10-13

**Authors:** Zhenzhu Song, Zhongchao Ma, Kai Qu, Sinan Liu, Wenquan Niu, Ting Lin

**Affiliations:** ^1^ Department of Clinical Laboratory, Liaocheng People’s Hospital, Taishan Medical College, Liaocheng 252000, China; ^2^ Department of Nephrology, Hemodialysis Center, Liaocheng People’s Hospital, Taishan Medical College, Liaocheng 252000, China; ^3^ Department of Hepatobiliary Surgery, The First Affiliated Hospital of Xi'an Jiaotong University, Xi’an 710061, China; ^4^ Department of Surgical Intensive Care Unit, The First Affiliated Hospital of Xi'an Jiaotong University, Xi’an 710061, China; ^5^ Project and Data Management Office, Institute of Clinical Medicine, China-Japan Friendship Hospital, Beijing 100029, China

**Keywords:** [TIMP-2] x [IGFBP7], acute kidney injury, meta-analysis, prediction

## Abstract

Acute kidney injury (AKI) most commonly occurs in critically ill and postoperative patients. Tissue inhibitor of metalloproteinases-2 (TIMP-2) and insulin-like growth factor-binding protein 7 (IGFBP7) are two newly-identified urinary biomarkers that can help to detect early AKI, yet their predictive accuracies range widely. Here, we conduct a systematic meta-analysis to evaluate the diagnostic values of [TIMP-2] x [IGFBP7] for AKI at different detection times and cutoff levels. Ten studies were meta-analyzed on 1606 patients. Overall, urinary [TIMP-2] x [IGFBP7] had a pooled sensitivity of 58% and specificity of 79%. Subgroup analysis showed that the sensitivity and specificity were 0.72 and 0.58 with a cutoff value of 0.3 (ng/mL)^2^/1000, and 0.38 and 0.94 with a cutoff value of 2.0 (ng/mL)^2^/1000, respectively. Moreover, when 0.3 was chosen as the cutoff value, restricting analysis to patients who were tested within 4 hours showed a sensitivity of 0.71 and specificity of 0.73, with the AUROC of 0.75. When 2.0 was chosen as the cutoff value, the sensitivity and specificity were 0.43 and 0.93, respectively in patients who were tested within 24 hours, with the AUROC of 0.70. In summary, urinary [TIMP-2] x [IGFBP7] can predict the occurrence of AKI with moderate diagnostic accuracy. In the earlier administrative periods (less than 4 hours), 0.3 (ng/mL)^2^/1000 is recommended to be used; whereas for patients who were administrated more than 24 hours, 2.0 (ng/mL)^2^/1000 is more appropriate.

## INTRODUCTION

Acute kidney injury (AKI) is a chief public health concern worldwide, and it is projected to affect 5% of patients admitted to the hospital and up to 50% of patients in intensive care units (ICU) [1]. Clinically, dehydration and sepsis combined with nephrotoxic drugs, especially following major surgeries, were considered the most common causes of AKI [2]. Currently, a growing concern has been paid to AKI, as it can precipitate the development of chronic kidney disease (CKD) and end-stage renal disease, and is associated with prolonged hospitalization and an increased mortality [3].

AKI ranks as one of the most expensive conditions, and in the United States the aggregative costs reached nearly $5–10 billion per year [1, 4]. Given that AKI is generally reversible and several therapies in animal models were proposed [5], to extend anti-AKI therapies from bench to bedside still has a long way to go. In view of the high prevalence of AKI and its deleterious consequences, it is clinically practical to identify certain accurate and reliable biomarkers to predict the occurrence of AKI.

Over the past decade, several urinary and blood biomarkers have been postulated for the early detection of AKI, including neutrophil gelatinase associated lipocalin (NGAL), kidney injury marker 1 (KIM-1) and interleukin-18 (IL-18), with inherent limitations [5, 6]. In pursuit of ideal biomarkers for AKI diagnosis, two cell-cycle arrest proteins, tissue inhibitor of metalloproteinases-2 (TIMP-2) and insulin-like growth factor-binding protein 7 (IGFBP7) have come to our sight. TIMP-2 and IGFBP7 were firstly identified in 2013 by Kashani *et al.* [7] and subsequently validated in the United States and European countries [8]. Afterwards, a growing number of studies have showed that the product of urinary TIMP-2 and IGFBP7, termed as [TIMP-2] x [IGFBP7] is a promising early indicator of AKI [9, 10]. However, when to detect this biomarker and how to select cutoff values thus far remains confused. To clear up this confusion, we attempted to evaluate the diagnostic value of urinary [TIMP-2] × [IGFBP7] for AKI through a meta-analysis based on recent clinical investigations.

## RESULTS

### Literature search

**Figure 1:**
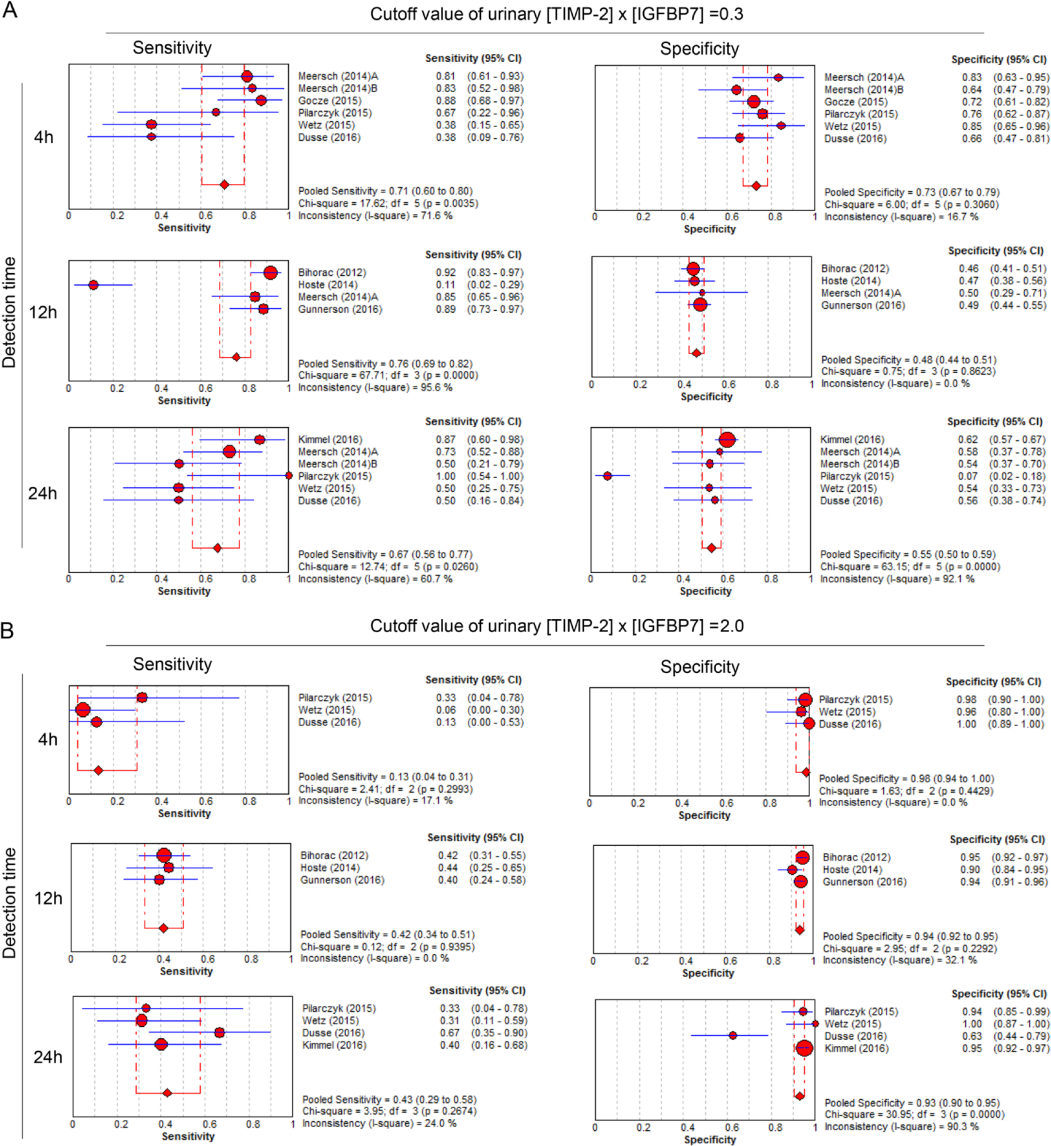
Forest plots of pooled sensitivities and specificities of urinary [TIMP-2] x [IGFBP7] for diagnosis of AKI at different cut off values and detection times (**A**) When the cutoff value of urinary [TIMP-2] x [IGFBP7] was 0.3, the pooled sensitivities and specificities were calculated at 4 h, 12 h and 24 h, respectively. (**B**) When the cutoff value of urinary [TIMP-2] x [IGFBP7] was 2.0, the pooled sensitivities and specificities were calculated at 4 h, 12 h and 24 h, respectively.

Figure 1 is a flow diagram that schematizes the selection of qualified articles in this meta-analysis. The initial literature search found 103 potentially relevant articles, and among them, 24 articles that seemingly met our inclusion and exclusion criteria were downloaded for further perusal. Finally, a total of 10 articles written in English and published between 2014 and 2016 were considered in this meta-analysis [11–20].

### Characteristics of included studies

The basic characteristics of 10 qualified studies with 1648 participants are shown in Table 1. There were three multicenter trials and five clinical trials that recruited more than 100 participants. Nine of ten studies (*n* = 1597) were conducted in adult participants, and one study (*n* = 51) in children [14]. Six studies (*n* = 350) were conducted to evaluate the diagnostic value of urinary [TIMP-2] x [IGFBP7] for AKI after major surgeries (5 cardiac surgery [13, 14, 16–18] and 1 non-cardiac surgery [15]), and four studies (*n* = 1298) were conducted among ICU patients [11, 12, 19, 20]. The definition of AKI in eight studies accorded with the Kidney Disease: Improving Global Outcomes classification (KDIGO) criteria. There was one study defining AKI according to the Risk, Injury, Failure, Loss of kidney function, and End-stage kidney disease (RIFLE) criteria [14] and the U-score [20], respectively.

**Table 1:** Basic characteristics of all eligible studies in this meta-analysis

Author (year)	Country	Study design	Clinical setting	Age (year)^a^	Male (%)^a^	*N*	History of CKD (%)^a^	AKI definition	Sampling time	Storage (°C)	Detection method
Bihorac (2014)	USA	Multicenter	Critically ill patients	62/63	49/55	408	7/8	KDIGO	within 12 h of ICU admission	-70	Nephro-Check test
Hoste (2014)	USA	Multicenter	Critically ill patients	64/65	44/60	153	15/7	KDIGO	within 12 h of ICU admission	-70	Nephro-Check test
Meersch (2014)A	Germany	Single center	After cardiac surgery	70/72	69.2/62.5	50	42.3/16.7	KDIGO	4 h/ 12 h/ 24 h after surgery	-70	Nephro-Check test
Meersch (2014)B	Germany	Single center	After pediatric cardiac surgery	1.5/3.0	16/57	51	NA	RIFLE	4 h/ 24 h after surgery	-80	Nephro-Check test
Gocze (2015)	USA	Multicenter	After non-cardiac surgery	60.03^b^	NA	107	15^b^	KDIGO	On ICU admission	NA	Nephro-Check test
Pilarczyk (2015)	Germany	Single center	After cardiac surgery	76.2/68.8	83.3/79.6	60	NA	KDIGO	4 h/ 24 h after surgery	NA	Nephro-Check test
Wetz (2015)	Germany	Single center	After cardiac surgery	75.0/66.5	NA	42	NA	KDIGO	End of surgery, 4 h/ 24 h after surgery	NA	Nephro-Check test
Dusse (2016)	Germany	Single center	After cardiac surgery	81.4/80.7	37.5/40.6	40	NA	KDIGO	4 h/ 24 h after surgery	NA	Nephro-Check test
Gunnerson (2016)	Europe and North America	Single center	Critically ill patients	67/64	66/64	375	9/11	KDIGO	within 12 h of ICU admission	NA	Nephro-Check test
Kimmel (2016)	Germany	Single center	Critically ill patients	68/66	80/66	362	7/11	U-Score	within 24 h of ICU admission	-70	Nephro-Check test

Abbreviations: CKD, chronic kidney disease; AKI, acute kidney injury; ICU, intensive care units; KDIGO, Kidney Disease: Improving Global Outcomes; RIFLE, Risk, Injury, Failure, Loss of kidney function, and End-stage kidney disease; NA, not available. ^a^ A backslash separating 2 values denotes AKI/no AKI. ^b^ Percentage in total patients.

The widely chosen time points of collecting urine samples were 4 h, 12 h and 24 h after surgery or ICU admission. All studies employed the commercial Nephro-Check kit to measure TIMP-2 and IGFBP7 in urine samples. Cutoff values for urinary [TIMP-2] × [IGFBP7] varied across studies. The widely used cutoff values were 0.3 and 2.0 (ng/mL)^2^/1000. Time of measurement, cutoff point and diagnostic accuracy of urinary [TIMP-2] × [IGFBP7] in each individual study for AKI diagnosis, including true positive (TP), true negative (TN), false positive (FP) and false negative (FN), sensitivity, specificity and the area under the summary receiver operating characteristic (AUROC) values are listed in Table 2.

**Table 2:** The diagnostic sensitivity and specificity of urinary [TIMP-2] x [IGFBP7] to predict AKI

Author (year)	Time of measurement	[TIMP-2]^*^ [IGFBP-7] cutoff value	TP	TN	FP	FN	Sensitivity	Specificity	AUROC (95% CI)
Bihorac (2012)	within 12 h of ICU	0.30	65	155	182	6	92.0%	46.0%	0.82 (0.76–0.88)
	admission	2.00	30	320	17	41	37.0%	95.0%	
Hoste (2014)	within 12 h of ICU	0.30	3	59	67	24	11.1%	46.8%	0.80 (0.74–0.84)
	admission	2.00	12	114	12	15	44.4%	90.5%	
Meersch (2014)A	4h after surgery	0.30	21	20	4	5	80.0%	83.0%	0.81 (0.68–0.93)
		0.40	16	21	3	10	62.0%	88.0%	
		0.50	14	22	2	12	54.0%	92.0%	
		0.60	12	22	2	14	46.0%	92.0%	
		0.70	12	24	0	14	46.0%	100.0%	
	12 h after surgery	0.30	22	12	12	4	85.0%	50.0%	NA
		0.40	20	18	6	6	77.0%	75.0%	
		0.50	13	20	4	13	65.0%	83.0%	
		0.60	15	22	2	11	58.0%	92.0%	
		0.70	14	22	2	12	54.0%	92.0%	
	24 h after surgery	0.30	19	14	10	7	73.0%	58.0%	0.90 (0.79–1.00)
		0.40	16	18	6	10	62.0%	75.0%	
		0.50	15	20	4	11	58.0%	83.0%	
		0.60	11	21	3	15	42.0%	88.0%	
		0.70	7	23	1	19	27.0%	96.0%	
Meersch (2014)B	4 h after surgery	0.30	10	25	14	2	83.0%	64.0%	0.85 (0.72–0.94)
		0.40	10	26	13	2	83.0%	67.0%	
		0.50	10	27	12	2	83.0%	69.0%	
		0.60	10	29	10	2	83.0%	74.0%	
		0.70	10	30	9	2	83.0%	77.0%	
	24 h after surgery	0.30	6	21	18	6	50.0%	54.0%	NA
		0.40	6	26	13	6	50.0%	67.0%	
		0.50	6	30	9	6	50.0%	77.0%	
		0.60	6	31	8	6	50.0%	79.0%	
		0.70	6	31	8	6	50.0%	79.0%	
Gocze (2015)	On ICU admission	0.30	21	60	23	3	86.7%	72.6%	0.85 (0.78–0.93)
Pilarczyk (2015)	4h after surgery	0.15	5	36	18	1	83.0%	66.7%	0.86 (0.72–1.00)
		0.30	4	41	13	2	67.0%	76.0%	
		2.00	2	53	1	4	33.0%	98.0%	
	24 h after surgery	0.89	5	44	10	1	80.0%	81.0%	0.82 (0.62–1.00)
		0.30	6	4	50	0	100.0%	7.0%	
		2.00	2	51	3	4	40.0%	95.0%	
Wetz (2015)	End of surgery	0.30	6	22	4	10	36.0%	84.0%	NA
		2.00	1	25	1	15	7.0%	96.0%	
	24 h after surgery	0.30	8	14	12	8	53.0%	54.0%	NA
		2.00	5	26	0	11	33.0%	100.0%	
Dusse (2016)	4 h after surgery	0.19	6	18	14	2	75.0%	56.0%	0.65 (0.38–0.92)
		0.30	3	21	11	5	38.0%	67.0%	
		2.00	1	32	0	7	13.0%	100.0%	
	24 h after surgery	0.30	4	18	14	4	55.0%	55.0%	0.87 (0.72–1.00)
		1.00	8	29	3	0	100.0%	90.0%	
		2.00	8	20	12	4	95.0%	62.0%	
Gunnerson (2016)	within 12 h of ICU	0.30	31	167	173	4	89.0%	49.0%	0.84 (0.76–0.90)
	admission	2.00	14	320	20	21	40.8%	94.0%	
Kimmel (2016)	within 24 h of ICU	0.30	13	215	2	87.0%	87.0%	62.0%	0.82 (0.66–0.93)
	admission	2.00	6	330	17	9	40.0%	95.0%	

Abbreviations: TIMP-2, tissue inhibitor of metalloproteinase-2; IGFBP7, insulin-like growth factor binding protein 7; TP, true positive; TN, true negative; FP, false positive; FN, false negative; AUROC, area under receiver operating characteristic curve; ICU, intensive care units; NA, not available.

### Comparison of diagnostic accuracies of different cutoff values and detection time

**Figure 2:**
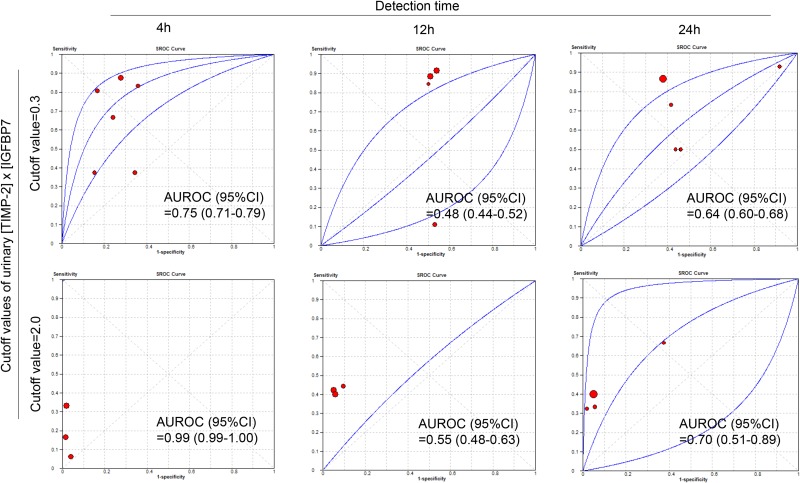
SROC curves of urinary [TIMP-2] x [IGFBP7] for diagnosis of AKI at different cut off values (0.3 or 2.0) and detection times (4 h, 12 h or 24 h)

The diagnostic accuracy of urinary [TIMP-2] × [IGFBP7] for AKI was also assessed across different subgroups. Table 3 summarizes the diagnostic accuracy of urinary [TIMP-2] × [IGFBP7] for AKI at cutoff values and detection times. At the cut-off values of 0.3 (ng/mL)^2^/1000, the summary sensitivities, specificities and AUROC were 0.72 (95% CI: 0.57–0.84), 0.58 (95% CI: 0.48–0.68) and 0.68 (95% CI: 0.64–0.73), respectively. Meanwhile, at the cut-off values of 2.0 (ng/mL)^2^/1000, the summary sensitivities, specificities and AUROC were 0.38 (95% CI: 0.19–0.42), 0.94 (95% CI: 0.93–0.95) and 0.75 (95% CI: 0.60–0.89), respectively (Table 3 and Figure 2). It is clearly shown that 0.3 (ng/mL)^2^/1000 had a relatively higher sensitivity and a lower specificity in diagnosing AKI, whereas, 2.0 (ng/mL)^2^/1000 had a lower sensitivity and a relatively higher specificity.

**Table 3:** Diagnostic accuracies of urinary [TIMP-2] x [IGFBP7] for AKI at different cutoff values and time points

Time of measurement ^a^	No. of studies	Sensitivity (95% CI)	Specificity (95% CI)	DOR(95% CI)	AUROC (95% CI)
**[TIMP-2] x [IGFBP7] = 0.3**					
Within 4 h	6	0.71 (0.60–0.80)	0.73 (0.67–0.79)	6.44 (2.72–15.2)	0.75 (0.71–0.79)
Within 12 h	4	0.76 (0.69–0.82)	0.48 (0.44–0.51)	2.65 (0.42–16.7)	0.48 (0.44–0.52)
Within 24 h	6	0.67 (0.56–0.77)	0.55 (0.50–0.59)	2.30 (0.94–5.63)	0.64 (0.60–0.68)
Overall	16	0.72 (0.57–0.84)	0.58 (0.48–0.68)	3.57 (1.77–7.21)	0.68 (0.64–0.72)
**[TIMP–2] x [IGFBP7] = 2.0**					
Within 4 h	3	0.13 (0.04–0.31)	0.98 (0.94–1.00)	8.59 (1.55–47.72)	0.99 (0.99–1.00)
Within 12 h	3	0.42 (0.34–0.51)	0.94 (0.92–0.95)	11.10 (7.02–17.6)	0.55 (0.48–0.63)
Within 24 h	4	0.43 (0.29–0.58)	0.93 (0.90–0.95)	8.33 (3.80–18.23)	0.70 (0.51–0.89)
Overall	10	0.38 (0.32–0.45)	0.94 (0.93–0.95)	10.2 (6.96–15.0)	0.75 (0.60–0.89)

Abbreviations: AKI, acute kidney injury; TIMP-2, tissue inhibitor of metalloproteinase-2; IGFBP7, insulin-like growth factor binding protein 7; DOR, diagnostic odds ratio; AUROC, area under receiver operating characteristic curve; NA, not available. ^a^Critically ill patients and patients after surgery were combined.

In addition, with prolonged sampling time, the sensitivity increased yet the specificity decreased. When 0.3 (ng/mL)^2^/1000 was chosen as the cutoff value of urinary [TIMP-2] × [IGFBP7] to predict AKI, the sensitivity and specificity were respectively 0.76 and 0.72 and the AUROC was 0.74 (95% CI, 0.70–0.78) in patients who were tested within 4 hours post-operatively (Table 4). When 2.0 (ng/mL)^2^/1000 was chosen as the cutoff value, the sensitivity and specificity were respectively 0.49 and 0.93 and the AUROC was 0.70 (95% CI, 0.51–0.89) in patients who were tested within 24 hours post-operatively (Table 4).

**Table 4:** The diagnostic sensitivity and specificity of urinary [TIMP-2] x [IGFBP7] to predict adverse kidney events^a^

Author (year)	Adverse kidney events	No of events/ total patients	Time of measurement	Cutoff value	TP	FP	FN	TN	Sensitivity	Specificity	AUROC (95% CI)
Dewitte (2015)	Death, need for RRT, or persistence of renal dysfunction at 30 days	16/57	On ICU admission	2.6	13	12	3	29	81%	71%	0.79 (0.66–0.88)
		16/57	At 24 h of ICU admission	2.3	9	1	7	40	57%	97%	0.81 (0.67–0.91)
Gocze (2015)	Need for RRT	10/107	On ICU admission	0.43	10	32	0	65	100%	67%	0.83 (0.75–0.92)
	Death in 28 days	10/107	On ICU admission	0.415	10	34	0	63	100%	64.9%	0.77 (0.67–0.86)
Westhoff (2015)	Need for RRT	16/46	On ICU admission	4.99	7	2	9	28	43.8%	93.6%	0.67 (0.50–0.84)
	Death in 30 days	6/46	On ICU admission	0.56	6	20	0	20	100%	50%	0.79 (0.61–0.97)

Abbreviations: RRT, renal replacement therapy; TIMP-2, tissue inhibitor of metalloproteinase-2; IGFBP7, insulin-like growth factor binding protein 7; TP, true positive; TN, true negative; FP, false positive; FN, false negative; AUROC, area under receiver operating characteristic curve; NA, not available.

^a^The definition of adverse kidney events was according to the National Institute of Diabetes and Digestive and Kidney Diseases (NIDDK) guideline [24].

### Diagnostic accuracy of urinary [TIMP-2] × [IGFBP7] for adverse kidney events

Adverse kidney events, including death, need for RRT, or persistence of renal dysfunction at 30 days were reported by only three studies [15, 21, 22]. In spite of inconsistent cutoff values in each study, we did not perform meta-analysis but only list their diagnostic accuracies of (Table 4). Data from those three studies showed that when detected urinary [TIMP-2] × [IGFBP7] on ICU admission, the lower cutoff values (less than 0.6) can be selected, with a high sensitivity (mean: 100%) and moderate specificity (mean: 61%).

## DISCUSSION

AKI is a common, complex disorder featured by a high mortality and costly complications. Previously, serum creatinine (Scr) and urine output are regarded as two “gold-standard” functional markers for the early detection of kidney injury, yet their diagnoses were later proven to be insensitive. In addition, Scr and urine output were also subject to confounding impact of muscle volume, diets and diuretics usage, which limited their further clinical application. To look for more sensitive and specific markers for renal injury, several clinical trials on AKI have been conducted and suggested some promising biomarkers, including NGAL, KIM-1, IL-18, liver-type fatty acid-binding protein (L-FABP) and cystatin C [6]. There is compelling evidence that NGAL was affected by age, renal function and severity of AKI, and NGAL and IL-18 can stimulate inflammation response [23]. Considering the fact that TIMP-2 and IGFBP7 are cell-cycle arrest proteins expressed in renal tubular cells during the periods of cellular stress or injury [8], it is tempting to speculate that urinary TIMP-2 and IGFBP7 are involved in the early pathological changes of AKI and carry the diagnostic probability of early renal injury. Now, several AKI cohort studies have evaluated the diagnostic value of the product of urinary TIMP-2 and IGFBP7, termed as [TIMP-2] × [IGFBP7] for AKI, whereas their detection times and cutoff values differed considerably. To fill this void in knowledge, we attempted to conduct a meta-analysis to quantify the appropriate detection time and cutoff value of urinary [TIMP-2] × [IGFBP7] for the early detection of AKI.

Via pooling the results of 10 clinical studies, our findings indicated that the performance of urinary [TIMP-2] × [IGFBP7] for predicting AKI was suboptimal, with a lower sensitivity (0.63; 95% CI, 0.49- 0.76) and a moderate specificity (0.76; 95% CI, 0.62- 0.86). Moreover, the AUC of 0.75 further suggested that the overall predictive accuracy was moderate.

It is a general practice to determine the optimal cutoff value of a continuous predictive marker. There are two widely used cutoff points for urinary [TIMP-2] × [IGFBP7] in the literature, viz. 0.3 and 2.0 (ng/mL)^2^/1000 in the assessment of AKI. In the Gunnerson’s study, the [TIMP-2] × [IGFBP7] > 0.3 (ng/mL)^2^/1000 had a sensitivity of 92% for moderate or severe AKI within the next 12 hours, and was associated with approximately 7 times risk compared with the [TIMP-2] × [IGFBP7] ≤ 0.3 (ng/mL)^2^/1000 [19]. However, the specificity of this cutoff point to detect AKI was only 46%. Therefore, it is not uncommon to encounter a false-positive finding if the test is used inappropriately in low-risk patients. The [TIMP-2] × [IGFBP7] value > 0.3 (ng/mL)^2^/1000 will have a greater predictive value in patients at high risk for AKI, but will be less useful in patients at low risk for AKI. A cutoff value of 2.0 (ng/mL)^2^/1000 for urinary [TIMP-2] × [IGFBP7] was associated with the specificity for moderate to severe AKI of 94%, although the sensitivity decreased to 38%.

In addition, we compared the diagnostic value of urinary [TIMP-2] × [IGFBP7] at different times for different cutoff value. It is of interest to note that with prolonged sampling time, the sensitivity increased yet the specificity decreased. It is reasonable to speculate that as the time went on, the secretion level of TIMP-2 and/or IGFBP7 increased gradually. So urinary [TIMP-2] × [IGFBP7] was low at the earlier time; if we choose a lower cutoff value at this time, we will derive a high specificity and a low sensitivity (compared at the same cutoff value). Further, the [TIMP-2] × [IGFBP7] becomes higher with prolonged time; if we choose a higher cutoff value at this time, we can derive a high sensitivity and a low specificity (compared at the same cutoff value). As such, we therefore developed a working hypothesis that if we set a low cutoff value, we should do the test earlier; if we set a high cutoff value, we should do the test later.

Several possible limitations in the present study merit serious consideration. First, with the purpose of avoiding low-quality studies, only published English articles were retrieved and articles in other languages was not covered, publication bias might be possible. Second, the types of participants in each study were various. Among of them, six studies were conducted among post-operative patients (5 studies cardiac surgery and 1 non-cardiac surgery), and four studies were conducted among ICU patients. It might cause significant heterogeneity between the selected studies. Therefore, the jury must refrain from drawing a conclusion until large, multi-center and well-performed clinical trials confirm or refuse our findings.

In summary, this meta-analysis provided evidence that urinary [TIMP-2] × [IGFBP7] can predict the occurrence of AKI with moderate diagnostic accuracy. In the earlier administrative periods (less than 4 hours), 0.3 (ng/mL)^2^/1000 is recommended to be used; whereas for patients who were administrated more than 24 hours, 2.0 (ng/mL)^2^/1000 is more appropriate. Nonetheless, it still remains an open question to determine the optimal time for urinary TIMP-2 and IGFBP7 measurement and the optimal cutoff value of [TIMP-2] × [IGFBP7] for the diagnosis of AKI. We hope further clinical trials with larger sample sizes and high-quality evidence are designed to clear away the clouds of these controversial issues convincingly.

## MATERIALS AND METHODS

### Data sources and search strategy

Two investigators (Ting Lin and Kai Qu) systematically and independently searched PubMed, EMBASE, Scopus and Web of Sciences databases for articles published before October 1, 2016 that provided data on the diagnostic accuracy of urinary [TIMP-2] × [IGFBP7] on the early identification of AKI in ICU patients. The conduct of this meta-analysis accorded with the Preferred Reporting Items for Systematic Reviews and Meta-Analyses (PRISMA) statement (Supplementary PRISMA checklist). Subject terms used for literature search embraced [“TIMP-2” OR “tissue inhibitor of metalloproteinase-2”] AND [“IGFBP7” OR “insulin-like growth factor binding protein 7”] AND [“acute kidney injury” OR “AKI”] AND [“diagnosis” or diagnostic”]. Search spectrum was also extended to the reference lists of retrieved original and review articles. Only studies with a prospective design and articles published in the English language were retained for analysis.

### Data extraction and synthesis

Two investigators (Ting Lin and Kai Qu) independently evaluated the study eligibility and quality according to the Quality Assessment of Diagnostic Accuracy Studies (QUADAS) score system. The data of interest were extracted on the first author, year of publication, population, study design, clinical setting, age, gender, history of CKD, AKI definition, time of measurement, urine sample storage and detection method. Diagnostic accuracy estimates included TP, FN, FN, TN. In addition, sensitivity, specificity, positive predictive value (PPV) and negative predictive value (NPV) for each reported test threshold were evaluated accordingly.

### Assessment of diagnostic test accuracy

The diagnostic test accuracy of the [TIMP-2] × [IGFBP7] for AKI diagnosis was quantified by the area under the summary receiver operating characteristic (SROC), summary diagnostic odds ratios (DORs) and summary sensitivities/specificities, respectively. Different SROC curves for the [TIMP-2] × [IGFBP7] were depicted and differences in SROC values were justified. The SROC curve delineates the relationship of the sensitivity against the specificity of different studies. Further summary DORs using a Der-Simonian and Laird random-effects model were computed, as well as between-study heterogeneity.

### Assessment of heterogeneity and publication bias

Between-study heterogeneity was represented by the Q-*I*^2^ statistic, which denotes the percentage of varied estimates accruing from heterogeneity rather than from sample errors. Significant heterogeneity was reported if the Q-*I*^2^ statistic is 50% or over. Potential contributing factors responsible for significant heterogeneity in the diagnostic accuracy of the [TIMP-2] × [IGFBP7] for AKI were sought by a meta-regression analysis. Also, heterogeneity was explored further by subgroup analyses across different clinical settings. In addition, the possibility of publication bias was visually inspected by the asymmetry of a Deek’s funnel plot in the prediction of urinary [TIMP-2] × [IGFBP7] for AKI.

Pooled sensitivity and specificity, DORs, and relevant 95% CIs were calculated on the basis of a bivariate normal model with log-transformed sensitivities and specificities. In the case of multiple cutoff points for the [TIMP-2] × [IGFBP7] provided in a single study, the point with the maximum overall accuracy entered into the overall analysis.

All statistical tests were two sided, and *P* < 0.05 was considered significant unless otherwise indicated. Above statistical analyses were completed with Stata 12.0.
